# Regional Variations in Physical Fitness in Children and Adolescents in Shaanxi Province

**DOI:** 10.3390/healthcare12181890

**Published:** 2024-09-20

**Authors:** Yanbing Li, Longhai Zhang, Shutong Yang, Ling Zhang, Jiaming Yan, Weixin Chen, Haiqi Zeng, Yuliang Sun, Wenfei Zhu

**Affiliations:** School of Physical Education, Shaanxi Normal University, Xi’an 710119, China; liyanbing@snnu.edu.cn (Y.L.); hae@snnu.edu.cn (L.Z.); yangshutong123@snnu.edu.cn (S.Y.); zhangling1@snnu.edu.cn (L.Z.); yjm1511094466@snnu.edu.cn (J.Y.); chenwxin@snnu.edu.cn (W.C.); zenghq@snnu.edu.cn (H.Z.)

**Keywords:** physical fitness, children and adolescents, geographical factors, regional variation

## Abstract

**Objectives:** This study aims to examine the geographical variation in physical fitness levels among Chinese children and adolescents in Shaanxi province. **Methods:** A total of 19,175 children from Shaanxi province with physical fitness data in 2019, participated in the study. Physical fitness was assessed using body mass index, force vital capacity, 50 m sprint, sit and reach, 1 min rope skipping, sit-ups, 50 m × 8 round-trip running, standing long jump, pull-ups, 800 m, and 1000 m running, and their standardized scores were aggregated to form a summary score. The total score is used to classify the physical fitness levels into four grades (excellence to failure). **Results:** The Guanzhong (GZ) region scored the highest, while Northern Shaanxi (NS) scored the lowest. The excellence rate for physical fitness was highest in GZ and lowest in NS, while the failure rate was highest in NS and lowest in GZ. Notably, children and adolescents in NS demonstrated the best endurance levels despite their overall lower fitness scores. The comprehensive physical fitness among Chinese children and adolescents in Shaanxi province showed significant regional disparities. GZ region exhibited the highest physical fitness levels, while Northern Shaanxi had the lowest. **Conclusions:** Region-specific interventions and targeted health policies are essential to address these disparities and improve the overall physical health status of children and adolescents in Shaanxi province.

## 1. Introduction

### 1.1. Present Condition of Physical Fitness

Physical fitness, defined as the body’s ability to perform activities effectively and efficiently [1], is a critical aspect of overall well-being and significantly contributes to leading a healthy and productive life [2]. For children and adolescents, physical fitness is particularly important as it enhances learning capabilities, promotes mental health, and cultivates healthy habits. Physical activity is a primary means to improve physical fitness [3]. In 2007, the Chinese government proposed that within about 5 years, the majority of adolescents should meet the national physical fitness standards, and promoted the Sunshine Sports Program in schools across the country [4]. In addition, the Chinese government has encouraged children to participate in sports through curriculum reforms and the improvement of public facilities, thereby promoting children’s physical fitness. A national study of Chinese children and adolescents found improvements in physical fitness from 2005 to 2014. However, children in the western provinces still exhibit lower fitness levels [1], largely due to natural environmental factors [5]. China issued the Outline of the Healthy China 2030 Plan in 2016, which includes specific requirements for children’s physical activity and fitness. By 2030, the plan mandates that all children and adolescents engage in at least one hour of physical activity daily, with more than 25% achieving an excellent level of physical fitness [6].

### 1.2. The Association between Geographic Factors and Physical Fitness

The association between geographic factors and physical fitness encompasses both the natural and built environments in which people live, as well as the socio-economic conditions that emerge from these environments. Numerous studies have confirmed the association between environmental changes and physical fitness across various geographical factors. The volume of research examining these links has been steadily increasing. Overall, previous research has focused on the built environment and socio-economic environment. Existing studies have shown that residence, sex, and ethnic disparities are associated with physical fitness and health to some extent [7,8,9,10]. Early research has demonstrated a correlation between children’s fitness and regional development, with economically advanced regions often showing better fitness levels among children and adolescents [5]. This disparity is frequently due to the unequal distribution of sports facilities across different areas. Some regions may have state-of-the-art stadiums and a wide range of exercise options, while others may lack adequate facilities, potentially reducing the motivation of children and adolescents to participate in physical activities [11]. Children living in economically developed regions receive more resources and financial support for physical activities. These regions tend to invest more in physical education. Thus, the better physical fitness levels among children in these regions [12,13]. Favorable natural environments motivate children and adolescents to engage in physical activity [14]. Poor natural environment has often been described as a barrier to physical activity. Extreme temperatures and excessive rainfall can greatly reduce the time children and adolescents spend on physical activities, which in turn can lead to a decline in their physical fitness [2,5,15,16]. Furthermore, poor air quality has been directly linked to decreased physical fitness levels in children [17,18]. The amount of green areas is positively associated with children’s mental health and immunity [19,20]. Thus, understanding the relationship between geographic environment and physical fitness is essential [21].

### 1.3. Regional Disparities in Physical Fitness across Shaanxi Province

China’s broad longitudinal and latitudinal range leads to diverse geographic, climatic, lifestyle, and economic characteristics among its regions. The Chinese government has implemented measures and policies to encourage physical activity among children and adolescents. While these initiatives have led to overall improvements in physical fitness levels, significant disparities in poor physical fitness persist in the western regions, including Shaanxi province [1]. 

As a major province in the western region, Shaanxi’s physical fitness levels are highly representative of the western regions. Shaanxi province, located in the center of China’s mainland, is divided into three distinct regions, each with significant differences in their natural geographic environments. The province spans three climate zones, leading to notable climatic variations between the northern and southern regions. The Guanzhong (GZ) region, known as the Central Shaanxi Plain, features flat plains and a warm temperate climate. The Qinling Mountains serve as the climatic divide between northern and southern China. Southern Shaanxi (SS), situated south of the Qinling Mountains, includes the Han River valley and is characterized by mountainous and forested terrain with a north subtropical climate. In contrast, the northernmost part of Shaanxi, near the Great Wall, is part of the Loess Plateau, known for its hilly and rugged terrain with extensive loess deposits, and experiences a mid-temperate climate [22].

### 1.4. Objectives

Natural geographic environmental factors influencing adolescent physical fitness often vary by region. However, research on these geographic associations in Shaanxi province is limited due to challenges in data acquisition. Moreover, there is currently no research specifically examining the differences in physical fitness levels among children and adolescents across the three distinct regions of Shaanxi province. This study aims to analyze the geographical variations in physical fitness among Chinese children and adolescents in Shaanxi province using 2019 data. By examining the spatial distribution and factors influencing physical health across different environments, we seek to clarify regional differences in physical fitness. The findings will help policymakers and health professionals design targeted public health measures for specific regions, identify disadvantaged areas, and ensure effective prioritization of resources under the national policy for children and adolescents’ physical health.

## 2. Materials and Methods

### 2.1. Study Population

The stratified whole cluster sampling method was employed for each city’s urban and rural areas. Four primary schools, two middle schools, and two high schools were selected in every city. All children and adolescents of these schools in the randomly selected classes were included in the survey, ensuring a minimum of 128 children and adolescents in each grade, evenly distributed between 64 boys and 64 girls. To ensure data accuracy, we conducted double data entry and eliminated any data that contained illogical values. A total of 19,715 individuals were included in the geographical variations analyses of physical fitness in this study, and informed consent was obtained from the children and adolescents and their parents.

### 2.2. Measurement of Physical Fitness

Participants in the survey underwent a complete physical fitness test according to the same protocol at all survey sites. All physical fitness tests were implemented in dedicated testing courses by trained staff who have passed the measurement examination. Following the Chinese National Survey on Students’ Constitution and Health (CNSSCH) handbook [23], the physical fitness projects include body mass index (BMI), force vital capacity (FVC), 50 m sprint, sit and reach, 1 min rope skipping, sit-ups, 50 m × 8 round-trip running, standing long jump, pull-ups, 800 m, and 1000 m running (Table 1). The Chinese National Student Physical Fitness Test Standard (2014 edition) [24] was used to test and score the physical fitness of all participants. All data were entered and checked twice by the experimenter, and the scores were checked to examine if they conformed to the logically defined values. Finally, scores were assigned to the measurements. The testing items and scoring standards vary across different grade levels.

Here is a summary of the testing methods for participants’ physical fitness:

(1) BMI test

The participant stands barefoot on a platform, and their weight is tested after hearing the prompt. Keep the heels, sacrum, and scapula in contact with the pillar. Maintain a natural upright posture with the head straight without looking down at the test data. Remember the two height and weight values, with only one test allowed. BMI = weight (kg)/height^2^ (m).

(2) FVC test

The participant uses a blowpipe and takes a deep breath until they can no longer inhale (avoid shoulder shrugging). Then, aim the mouth at the force vital capacity-blowing mouthpiece and exhale deeply at a steady pace without pausing until the air is fully exhaled. Data are locked as soon as exhalation is complete. Multiple tests can be conducted if needed.

(3) Sit and reach test

The participant sits on a flat surface with legs extended and feet about 10–15 cm apart. Bend forward at the waist with both arms reaching forward evenly to push the slider steadily without sudden force until they cannot push further. Maintain straight legs without bending.

(4) Standing long jump test

The participant stands naturally with both feet apart at a starting line, ensuring no foot touches or crosses the line. Jump as far as possible from a stationary position without stepping or hopping. Perform a total of three tests and record the best result.

(5) Pull-ups (for boys) and Sit-ups (for girls)

Pull-ups: The participant grips the bar with palms facing forward, arms shoulder-width apart in a straight-arm hang. Pull up simultaneously with both arms, lifting the chin above the bar’s upper edge for one repetition.

Sit-ups: The participant lies on a mat, knees bent at a 90-degree angle, arms crossed behind the head. Complete as many sit-ups as possible within one minute, ensuring the elbows touch or go beyond the knees. The shoulders must touch the mat during the downward phase.

(6) 50 m run

Conducted in groups of at least two individuals. Participants stand at the starting line and begin running upon hearing the command “Go”. The starter simultaneously waves a flag to give the command. A timer starts measuring time the moment the flag is waved, and timing stops when the participant’s torso crosses the vertical plane of the finish line.

(7) 1000 m run (for boys)/800 m run (for girls)

After verification of identity by registration personnel and receiving numbered middle-distance running vests, participants are divided into groups of 20 to 30 individuals. They are then taken to the starting point. The starting line judge instructs the participants, guides them to stand in a stationary position, and gives the command, “On your marks, get set, go” to initiate the run. The timer starts measuring the time at the sound of the starting signal, and the judge records the participant’s final score when they reach the finish line. 

Considering the varying items for boys and girls across different age groups and the challenges in making direct comparisons, sex- and age-specific standardized values were calculated for each physical fitness component. The physical fitness score was calculated by first determining the score for each indicator based on its assigned weight and then summing all these individual scores to obtain the overall physical fitness score. Based on the physical fitness score, we categorized it into four levels: Excellent (≥90), Good (<90 and ≥80), Pass (<80 and ≥60), and Failure (<60).

## 3. Statistical Analysis

The descriptive statistics are reported as means, standard deviations, frequencies, and percentages as appropriate. A one-way analysis of variance (ANOVA) was conducted to examine variations in continuous variables in physical fitness indexes. When significant differences were found, post-hoc paired *t*-tests were conducted to explore regional differences, with Bonferroni correction applied to account for multiple comparisons. The Chi-square tests were used to compare frequencies between groups’ differences in categorical variables. 

Pearson’s correlation coefficients were used to examine the relationships between geographical variables and overall physical fitness scores. The hierarchical multiple regression was conducted to identify the predictors of physical fitness. Statistical significance was set at *p* < 0.05. All statistical analyses were conducted with SPSS 25.0 (IBM, Chicago, IL, USA).

## 4. Results

The current study comprised a total of 19,715 participants, 9480 (49.91%) of whom were boys and 9875 (50.09%) of whom were girls. There are 8620 (43.72%) participants from GZ, including 4305 (21.84%) boys and 4315 (21.89%) girls. There are 4482 (22.73%) participants from NS, including 2279 (11.56%) boys and 2206 (11.19%) girls. There are 6610 (33.53%) participants from SS, including 3256 (16.52%) boys and 3354 (17.01%) girls.

### 4.1. Geographical Environmental Factors

All the schools included in this study are located in Shaanxi province, China, covering all cities. To examine geographical variation, the ten mainland cities in Shaanxi were divided into three regions based on geographical standards: GZ (including Xi’an, Xianyang, Weinan, Tongchuan, and Baoji), NS (including Yulin and Yan’an), and SS (including Hanzhong, Ankang, and Shangluo) [25]. Geographical data were sourced from the 2020 Annual Statistical Report provided by the Shaanxi Provincial Bureau of Statistics. Table 2 illustrates the differences in geographical environmental factors among GZ, NS, and SS. These factors include average temperature, annual sunshine hours, relative humidity, precipitation, average altitude, and green area per capita.

Significant differences in average temperature were observed among the three regions (F = 8.63, *p* = 0.013). SS had the highest average temperature (15.03 ± 1.46 °C), followed by GZ (13.82 ± 1.40 °C), and NS with the lowest (10.15 ± 0.21 °C). Annual sunshine hours also showed significant differences (F = 14.41, *p* = 0.003). NS received the most sunshine (2631.75 ± 275.14 h), followed by GZ (1887.60 ± 249.86 h), and SS had the least (1372.80 ± 261.31 h). Relative humidity varied significantly among the regions (F = 10.14, *p* = 0.009). SS had the highest relative humidity (71.00 ± 4.58%), followed by GZ (64.20 ± 1.92%), with NS having the lowest (50.00 ± 11.31%). The average altitude showed significant differences (F = 8.89, *p* = 0.012). NS had the highest altitude (1081.00 ± 22.63 m), followed by SS (501.33 ± 230.26 m), and GZ had the lowest (489.40 ± 166.40 m). No significant differences were observed in precipitation (F = 2.89, *p* = 0.122) and green area per capita (F = 2.88, *p* = 0.120) among the three regions.

### 4.2. The Differences in Physical Fitness Across Different Region Groups

The results indicated significant regional differences in the physical fitness of children and adolescents in Shaanxi province (Table 3 and Table 4). 

BMI was slightly lower in SS compared to GZ and NS (F = 12.53, *p* < 0.001). GZ had the highest FVC values, followed by SS, with NS showing the lowest (F = 76.34, *p* < 0.001). In the 50 m sprint, participants from GZ were the fastest, followed by those from SS, while NS had the slowest times (F = 27.07, *p* < 0.001).

As measured by the sit and reach test, flexibility was greatest in GZ, followed by SS, with NS exhibiting the least flexibility (F = 234.86, *p* < 0.001). In the 1 min rope skipping test, GZ participants performed best, with SS in the middle and NS performing the least (F = 933.95, *p* < 0.001). Similarly, in the 1 min sit-up test, children from GZ completed the most sit-ups, followed by those from SS, with NS children doing the fewest (F = 142.58, *p* < 0.001).

The 50 m × 8 round-trip running results showed GZ had the fastest times, followed by SS, while NS participants were the slowest (F = 76.90, *p* < 0.001). The standing long jump results were slightly lower in NS compared to GZ and SS (F = 92.98, *p* < 0.001). In the pull-ups test, SS participants performed the most pull-ups, followed by GZ, with NS performing the fewest (F = 14.85, *p* < 0.001).

Endurance tests, including the 800 m and 1000 m runs, revealed that NS participants recorded the fastest times, followed by SS, with GZ being the slowest in both events (800 m: F = 87.82, *p* < 0.001; 1000 m: F = 344.47, *p* < 0.001). Lastly, the overall physical fitness score was highest in GZ, followed by SS, with NS scoring the lowest (F = 486.56, *p* < 0.001).

### 4.3. Distribution of Physical Fitness Levels among Boys and Girls in Shaanxi Province

**Figure 1:**
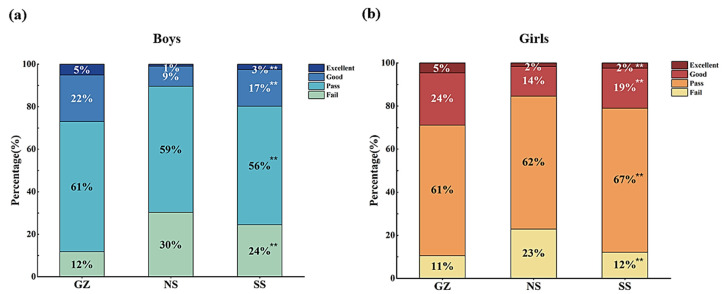
Percentage of different levels of physical fitness among boys and girls in three regions of Shaanxi province. (**a**) The percentage of each of the physical fitness levels of boys; (**b**) the percentage of each of the physical fitness levels of girls; GZ: Guanzhong; NS: North Shaanxi; and SS: South Shaanxi. **: A statistically significant difference in the proportions of each level of physical fitness using Chi-square tests (*p* < 0.001).

Figure 1 illustrates the distribution of physical fitness levels among boys and girls across three regions in Shaanxi province. Among the boys, significant differences were observed in the “Failure” category (*p* < 0.001). NS has the highest percentage of boys failing and GZ has the lowest. Significant differences were found in the “Pass” category (*p* < 0.001), with GZ having the highest percentage and NS the lowest. Significant differences were observed in the “Good” category (*p* < 0.001), with GZ having the highest percentage and NS the lowest. Similarly, significant differences were noted in the “Excellent” category (*p* < 0.001), where GZ also had the highest percentage of boys, and NS the lowest.

Among the girls, significant differences were observed in the “Failure” category (*p* < 0.001), with NS recording the highest percentage of girls failing and GZ the lowest. Significant differences were also observed in the “Pass” category (*p* < 0.001), where SS had the highest percentage of girls passing, and GZ had the lowest. Significant differences were observed in the “Good” category (*p* < 0.001), with GZ showing the highest percentage of girls and NS the lowest. Additionally, significant differences were found in the “Excellent” category (*p* < 0.001), with GZ again having the highest percentage of girls and NS the lowest. 

### 4.4. Correlation Analysis of Physical Fitness Scores and Geographical Variations

Correlations between overall physical fitness scores and other key variables are presented in Table 5. Physical fitness scores were positively correlated with sex (r = 0.077, *p* < 0.001), average temperature (r = 0.072, *p* < 0.001), and green area per capita (r = 0.072, *p* < 0.001). Conversely, physical fitness scores were negatively correlated with grades (r = −0.223, *p* < 0.001), regions (r = −0.129, *p* < 0.001), annual sunshine hours (r = −0.036, *p* < 0.001), relative humidity (r = −0.017, *p* = 0.039), precipitation (r = −0.139, *p* < 0.001), and average altitude (r = −0.089, *p* < 0.001).

### 4.5. Hierarchical Regression Analysis of Physical Fitness Predictors

To identify key predictors of physical fitness, we conducted a hierarchical multiple regression analysis (Table 6).

We added sex, grades, and regions. This model was statistically significant, F_(3, 19711)_ = 583.32, *p* < 0.001, and accounted for 7.5% of the variance in physical fitness scores. Each of the factors significantly predicted physical fitness scores: sex (β = 1.86, *p* < 0.001), grades (β = −0.35, *p* < 0.001), and regions (β = −1.86, *p* < 0.001).

The hierarchical multiple regression analysis indicated that significant differences in physical fitness among the three regions remained evident even after adjusting for age and grade. Post-hoc Bonferroni tests further confirmed significant differences between GZ and NS (*p* < 0.001, 95% CI [4.20, 5.19]) as well as between SS and NS (*p* < 0.001, 95% CI [−5.51, −4.47]). In contrast, no significant difference was observed between GZ and SS (*p* = 0.319, 95% CI [−7.32, 0.14]).

## 5. Discussion

This is the first study that used cross-sectional surveys to assess the geographic variations in comprehensive physical fitness among children and adolescents in Shaanxi province. The results revealed notable geographic variability, showing that children and adolescents in the GZ region have better physical fitness levels compared to those in the NS and SS regions. Thus, this study highlights the importance of considering regional factors when developing targeted interventions to improve adolescent physical fitness in Shaanxi province.

The study found that girls generally have better physical fitness than boys among children and adolescents in Shaanxi province. Boys generally excelled over girls in metrics such as FVC, speed tests, and strength activities. These findings align with the existing literature, which indicates that boys typically have higher aerobic capacity and muscular strength than girls, likely due to physiological differences [26]. Additionally, higher grade levels are associated with poorer physical fitness, likely due to increased academic pressures and recreational screen time [27]. These pressures result in more sedentary time and reduced opportunities for participating in sports activities [28].

The analysis revealed that boys and girls from the GZ region generally outperformed their peers from the SS and NS regions in most physical fitness metrics. The NS region exhibited the poorest physical fitness levels. These differences can be attributed to several factors. Temperature plays a significant role [29]. Our study found that higher average temperatures are correlated with better physical fitness. GZ’s moderate climate, characterized by mild temperatures, facilitates year-round physical activity without the hindrance of extreme weather conditions. In contrast, NS has a semi-arid climate with long, cold winters, often featuring strong winds and temperatures well below freezing. Such harsh conditions pose potential injury risks, discouraging adolescents from participating in physical activities. This finding is consistent with studies from Norway and the UK, which showed increased physical activity in warmer temperatures and decreased activity in colder temperatures [30,31]. The population in warmer regions may take advantage of favorable conditions to engage in more physical activity. Compared to cold and dry weather, individuals may engage in physical activity more frequently in warm and humid environments [2]. Additionally, a previous study in Hong Kong found a negative association between temperature and physical activity [32], possibly due to Hong Kong’s higher temperatures compared to Shaanxi province [16].

Extreme heat has been recognized as a deterrent to outdoor activities. Our study found that the NS region has the longest annual sunshine hours, which are correlated with poorer physical fitness. Extended periods of sunshine lead to higher temperatures and increased ultraviolet radiation, which not only raises the risk of skin cancer [33] but also reduces the time individuals spend on physical activities [34]. These findings are quite similar to those of previous studies. A study conducted in Galveston indicated that physical activity levels were lower in higher temperatures compared to cooler weather. This is attributed to the fact that in Galveston, high temperatures can reach up to 29 °C (84 °F) [35]. Another study found that physical activity levels decrease in cities with high temperatures compared to those with warm climates [16].

Green areas per capita also were identified as a positive predictor of physical fitness. Our study found that the green area per capita in the GZ region is superior to that in SS and especially NS, which are correlated with better physical fitness. These findings are consistent with studies from the Yangtze River Delta urban agglomerations, which show that green space per capita has a significant positive association on public health levels [36]. The availability of green spaces provides safe and accessible locations for physical activities such as running, playing sports, and other exercises [36]. An increase in the amount of green space will have a beneficial association with the health of people [37].

Our study found that the SS region has the highest humidity and rainfall, while the NS region experiences the lowest. These factors—high relative humidity and precipitation—are associated with poorer physical fitness levels. The SS region, influenced by the Qinling Mountains, experiences higher temperatures and more rainfall in the summer, especially during summer and autumn. This makes it the most humid area in Shaanxi province, which may to some extent affect the physical activity of adolescents and children in this region. This result is consistent with the previous study, a scoping review from 2006 to 2020 which proved that associations between humidity and precipitation and physical activity volume were positively negative [38]. Recent research has emphasized the significant association of annual precipitation with the physical fitness of children and adolescents. The amount of rainfall contributes to a moist environment, improved air quality, and reduced dust and pollution, creating favorable conditions for physical activity. However, Excessive rainfall can lead to muddy or slippery playgrounds and sports fields, rendering them unsuitable for physical activities and potentially disrupting children and adolescents’ exercise routines [5,15]. Sunlight helps in the synthesis of vitamin D, which promotes bone health [2]. It is important to note that inadequate daylight hours, particularly during gloomy seasons, may adversely affect mood and motivation, diminishing the desire for outdoor activities. 

Changes in the natural environment brought about by altitude can affect the human body in a variety of ways [5]. Poor air quality has decreased physical fitness in children [17,18], high-altitude areas often experience lower oxygen levels and higher ultraviolet radiation [34], which can also reduce the amount of time individuals engage in physical activities. Our study found that altitude is linked to poorer physical fitness levels. NS located on the Loess Plateau, has an average altitude of 1020 m—the highest among the three regions. Previous studies have shown that for each 1 m increase in altitude, the rate of low physical fitness in each province decreased by an average of 0.552% [5]. The physical fitness of children and adolescents in high-altitude areas of Tibet is generally lower than that in low-altitude regions [34]. Although the overall physical fitness of children and adolescents in the NS region is the poorest, their performance is better in aerobic capacity compared to those in GZ and SS. This performance may be directly related to the region’s high altitude and unique geographic characteristics. As altitude increases, the oxygen levels gradually decrease. Compared to GZ and SS, children in the NS region have long-term exposure to a relatively low-oxygen environment. This environment enhances their oxygen-carrying capacity leading to the production of red blood cells [39], and increases total hemoglobin mass [40] providing natural high-altitude training benefits [41]. A similar previous study demonstrated significant differences in physical fitness performance among Tibetan adolescents at various altitudes, with those at higher altitudes showing superior endurance and cardiovascular performance [34]. This aligns with our observations in NS, indicating the role of high-altitude environments in enhancing aerobic fitness.

The geographical and climatic differences among GZ, NS, and SS significantly influence the lifestyle, and overall living conditions in each region. These factors, in turn, affect the physical fitness and health levels of the population, highlighting the importance of region-specific health and fitness interventions. Therefore, maintaining appropriate levels of precipitation, temperature, and annual sunshine hours is vital for creating an optimal outdoor exercise environment, encouraging children and adolescents’ participation in sports activities, and ultimately benefiting their physical health. However, it is essential to acknowledge that these factors can vary significantly across regions and seasons, necessitating the consideration of local meteorological conditions when planning physical activities. Moreover, schools and physical education institutions should implement necessary safety measures to ensure that children and adolescents can safely engage in exercise even during unfavorable weather conditions.

This study has several advantages. First, the study effectively correlates the physical fitness and health of children and adolescents with geographic environmental factors, shedding light on the relationship between geographic distribution characteristics and influencing factors. Second, by including children and adolescents of different grades from various cities in Shaanxi province, the study ensures a broad and diverse sample. This enhances the reliability and representativeness of the research results. Third, this study conducts a comprehensive physical fitness assessment of children and adolescents, evaluating respiratory function, strength, flexibility, explosive power, and cardiorespiratory endurance. By monitoring these diverse aspects of physical fitness, the study provides a holistic view of the children’s health and fitness levels. This comprehensive approach allows for a more detailed and accurate assessment, which is crucial for developing effective interventions and health policies. Additionally, the use of multiple fitness indicators ensures that various dimensions of physical fitness are thoroughly evaluated, highlighting the multifaceted nature of physical health in young populations.

This study also has some disadvantages. First, while it identifies the association between geographic and environmental factors and the physical fitness and health of children and adolescents, it does not explore the specific mechanisms behind these factors in detail. Further research is needed to explore why children and adolescents in central regions have higher physical fitness scores and whether this is related to factors such as the region’s economic level and cultural background. Second, the study lacks long-term follow-up to observe trends in physical fitness among children and adolescents. Conducting longitudinal studies would help better understand the effects and influencing factors of interventions over time, providing more comprehensive insights. Third, the study does not adequately address the impact of family culture, socio-cultural factors, and other geographic influences on physical fitness and health. Further in-depth research is needed to evaluate these associations and develop targeted interventions. By understanding these factors, more effective physical fitness policies and educational strategies can be devised to improve the health status of children and adolescents. 

## 6. Conclusions

The findings from this study revealed the characteristics and differences in the spatial distribution of children and adolescents’ physical health in Shaanxi province in association with the geographic environment. Compared to children living in the NS and SS regions, children living in the GZ region have better physical fitness levels. The study identified correlations between physical fitness and factors such as average temperature, annual sunshine hours, relative humidity, precipitation, average altitude, and green area per capita, indicating that these geographic factors can predict physical fitness levels across different regions. These insights suggest that strategies aimed at improving physical fitness in Shaanxi province should take into account the unique geographical characteristics of each region. Policies should be tailored to address geographic disparities and promote physical fitness more effectively across diverse areas. 

## Figures and Tables

**Table 1:** The content of the test and the weighting of the scores for each academic level.

Test Subject	Individual Indicators	Weights%
Primary 1 to High School	BMI	15
	FVC	15
Primary 1 and 2	50 m running	20
	Sit and reach	30
	1 min rope skipping	20
Primary 3 and 4	50 m running	20
	Sit and reach	20
	1 min rope skipping	20
	1 min sit-ups	10
Primary 5 and 6	50 m running	20
	Sit and reach	10
	1 min rope skipping	10
	1 min sit-ups	20
	50 m × 8 round-trip running	10
Junior High School, and High School	50 m running	20
	Sit and reach	20
	Standing long jump	10
	1 min pull-ups (Boys)/1 min sit-ups (Girls)	10
	1000 m running (Boys)/800 m running (Girls)	20

**Table 2:** Differences in geographical environmental factors between the three regions (M ± SD).

	GZ		NS		SS		F	*p*
	M	SD	M	SD	M	SD		
Average temperature (°C)	13.82 ^a^	1.40	10.15 ^c^	0.21	15.03	1.46	8.63	0.013 *
Annual sunshine hours (h)	1887.60 ^a^	249.86	2631.75 ^c^	275.14	1372.80	261.31	14.41	0.003 *
Relative humidity (%)	64.20 ^a^	1.92	50.00 ^c^	11.31	71.00	4.58	10.14	0.009 *
Precipitation (mm)	650.22	115.11	610.90	119.50	882.13	207.05	2.89	0.122
Average altitude (m)	489.40 ^a^	166.40	1081.00 ^c^	22.63	501.33	230.26	8.89	0.012 *
Green area per capita (hectare)	23.01	10.79	8.57	1.50	16.80	2.69	2.88	0.120

GZ: Guanzhong; NS: North Shaanxi; SS: South Shaanxi; ^a^: GZ vs. NS; ^c^: NS vs. SS; and * *p* < 0.05.

**Table 3:** The difference in physical fitness in children and adolescents in Shaanxi province (M ± SD).

	GZ (*n* = 8622)		NS (*n* = 4484)		SS (*n* = 6609)		F	*p*
	M	SD	M	SD	M	SD		
BMI (kg/m^2^)	19.37	3.78	19.44	3.92	19.11	3.75	12.53	<0.001 **
FVC (mL)	2447.55	1138.49	2235.04	1005.09	2269.56	1085.04	76.34	<0.001 **
50 m running (s)	9.59	1.43	9.80	1.78	9.68	1.51	27.07	<0.001 **
Sit and reach (cm)	35.36	32.81	22.70	28.65	31.80	32.55	234.86	<0.001 **
1 min rope skipping (Primary School Grades 4–6)	95.35	38.76	55.12	36.08	72.30	37.25	933.95	<0.001 **
1 min sit-ups (Primary School Grades 4–6)	28.63	9.85	24.39	10.37	26.97	10.55	142.58	<0.001 **
50 m × 8 round-trip running (s) (Primary School Grades 5–6)	117.43	13.19	125.06	16.17	119.43	14.47	76.90	<0.001 **
Standing long jump (cm) (Junior High School, and High School)	177.90	33.78	163.75	32.79	177.95	34.74	92.98	<0.001 **
Pull-ups (Junior High School, and High School)	2.08	3.43	2.02	3.72	2.74	3.83	14.85	<0.001 **
800 m running (s) (Junior High School, and High School)	260.26	29.10	235.03	56.51	250.29	30.06	87.82	<0.001 **
1000 m running (s) (Junior High School, and High School)	280.84	44.11	232.90	68.77	266.91	36.99	344.47	<0.001 **
Overall physical fitness score	73.73	11.31	67.22	11.84	70.55	11.58	486.56	<0.001 **

GZ: Guanzhong; NS: North Shaanxi; SS: South Shaanxi; BMI: body mass index; FVC: force vital capacity; ** *p* < 0.001.

**Table 4:** Bonferroni multiple comparison analysis of differences in physical fitness among children and adolescents.

	GZ vs. NS				GZ vs. SS				NS vs. SS			
	*t*	*p*	95%CI		*t*	*p*	95%CI		*t*	*p*	95%CI	
			LCL	UCL			LCL	UCL			LCL	UCL
BMI (kg/m^2^)	−1.01	0.918	−0.24	0.10	4.13	<0.001 **	0.11	0.41	4.46	<0.001 **	0.15	0.51
FVC (mL)	10.57	<0.001 **	164.39	260.62	−9.97	<0.001 **	135.26	220.71	−10.57	0.306	−85.08	16.03
50 m running (s)	−7.30	<0.001 **	−0.28	−0.14	−3.59	0.001 *	−0.15	−0.03	3.92	<0.001 **	0.05	0.19
Sit and reach (cm)	21.61	<0.001 **	11.26	14.07	6.85	<0.001 **	2.32	4.81	−14.78	<0.001 **	−10.57	−7.63
1 min rope skipping (Primary School Grades 4–6)	41.82	<0.001 **	37.93	42.54	26.96	<0.001 **	21.00	25.09	−17.30	<0.001 **	−19.57	−14.81
1 min sit-ups (Primary School Grades 4–6)	16.83	<0.001 **	3.64	4.85	7.60	<0.001 **	1.14	2.18	−9.80	<0.001 **	−3.22	−1.96
50 m × 8 round-trip running (s) (Primary School Grades 5–6)	−12.27	<0.001 **	−9.13	−6.14	−3.57	<0.001 **	−3.34	−0.66	8.87	<0.001 **	4.12	7.16
Standing long jump (cm) (Junior High School, and High School)	13.07	<0.001 **	11.55	16.74	−0.06	1.000	−2.10	2.00	−12.07	<0.001 **	−17.01	−11.38
Pull-ups (Junior High School, and High School)	0.04	1.000	−0.32	0.43	−0.51	<0.001 **	−0.98	−0.35	−0.42	<0.001 **	−1.14	−0.30
800 m running (s) (Junior High School, and High School)	12.90	<0.001 **	13.56	19.75	7.98	<0.001 **	6.98	12.97	−4.81	<0.001 **	−10.01	−3.35
1000 m running (s) (Junior High School, and High School)	26.22	<0.001 **	43.56	52.32	8.45	<0.001 **	9.98	17.88	−17.29	<0.001 **	−38.72	−29.30
Overall physical fitness score	30.70	<0.001 **	6.01	7.02	1.69	<0.001 **	2.74	3.64	−14.93	<0.001 **	−3.86	−2.79

GZ: Guanzhong; NS: North Shaanxi; SS: South Shaanxi; BMI: body mass index; FVC: force vital capacity; LCL: lower limit of the confidence interval; UCL: upper limit of the confidence interval: ** *p* < 0.001; and * *p* < 0.05.

**Table 5:** Correlations between overall physical fitness scores and other variables.

Variables	1	2	3	4	5	6	7	8	9	10
1. Overall physical fitness score	1.00									
2. Sex ^a^	0.08 **	1.00								
3. Grades	−0.22 **	0.01	1.00							
4. Regions ^b^	−0.13 **	0.01	−0.03 *	1.00 **						
5. Average temperature	0.07 **	0.01 *	0.00	0.21 **	1.00 **					
6. Annual sunshine hours	−0.04 **	−0.01	0.00	−0.40 **	−0.93 **	1.00 **				
7. Relative humidity	−0.02 *	0.01	0.01	0.29 **	0.77 **	−0.89 **	1.00 **			
8. Precipitation	−0.14 **	0.01	0.02 *	0.58 **	0.64 **	−0.78 **	0.69 **	1.00 **		
9. Average altitude	−0.09 **	−0.01 *	0.00	0.04 **	−0.92 **	0.78 **	−0.70 **	−0.43 **	1.00 **	
10. Green area per capita	0.07 **	−0.01 *	−0.02 *	−0.19 **	−0.54 **	0.53 **	−0.49 **	−0.47 **	0.46 **	1.00 **

Note: ^a^ 0 = boy,1 = girl; ^b^ 1 = Guanzhong, 2 = North Shaanxi, 3 = South Shaanxi; ** *p* < 0.001; and * *p* < 0.05.

**Table 6:** Hierarchical multiple regression summary predicting physical fitness scores.

Predictor Variable	β	*t*
Sex ^a^	1.86	11.49 **
Grades	−0.35	−33.30 **
Regions ^b^	1.86	−20.01 **
*R* ^2^	0.075	
*1* *R*^2^	0.075	
*F*	530.32	

Note: ^a^ 0 = boy, 1 = girl; ^b^ 1 = Guanzhong, 2 = North Shaanxi, 3 = South Shaanxi; and ** *p* < 0.001.

## Data Availability

The data presented in this study are available on request from the corresponding author. The data are not publicly available due to confidentiality.
